# Monitoring patients on ART within the CCMDD programme and those attending an urban healthcare facility in KwaZulu-Natal

**DOI:** 10.4102/safp.v66i1.5972

**Published:** 2024-10-23

**Authors:** Sheldon Chetty, Andrew Ross

**Affiliations:** 1 East Boom Community Health Centre, Department of Health, Pietermaritzburg, South Africa; 2 Department of Family Medicine, Faculty of Health Science, University of KwaZulu-Natal, Durban, South Africa

**Keywords:** HIV, CCMDD, decongestion of urban healthcare facilities in Pietermaritzburg, KwaZulu-Natal, viral load, CCMDD, decongestion of healthcare facilities, overburdened health facilities, virologically stable, decanting of patients, differentiated model of care

## Abstract

**Background:**

South Africa has high number of patients on antiretroviral treatment, necessitating innovative approaches to decongest healthcare facilities. The Central Chronic Medicines Dispensing and Distribution (CCMDD) programme is a national initiative that identifies stable chronic patients for collection at pick-up points away from the health facility. This study aimed to compare patient satisfaction and virological suppression among those who collected medication through the CCMDD programme and routine care.

**Methods:**

This descriptive retrospective analytical study was conducted at a community health centre in Pietermaritzburg from 01 January 2018 to 31 December 2018 and included a questionnaire and access to their medical records on the national medicines database. The 117 patients in the routine care and CCMDD programme groups were assessed at baseline and evaluated at 6 months and 12 months, which were the time points for viral load (VL) testing.

**Results:**

Of the 234 participants, 34 out of 117 (31.6%) remained in routine care at the 6-month review, and all but 7 patients had transferred to the CCMDD after 12 months. At the end of the study, 7 patients had VLs above 50 copies/mL and continued in routine care, while 97% (*n* = 27/234) remained virologically suppressed. None of the CCMDD programme patients moved out of the programme.

**Conclusion:**

Satisfaction with the CCMDD programme is indicated by the patients’ continued VL suppression, highlighting its potential to decongest healthcare facilities and reduce the strain associated with medication collection.

**Contribution:**

The findings in this study validate patients being registered onto the CCMDD programme.

## Introduction

South Africa has the largest antiretroviral programme in the world,^1,2,3^ with the Department of Health introducing universal access to antiretroviral therapy (ART) for patients living with human immunodeficiency virus (HIV) and acquired immunodeficiency syndrome (AIDS) in September 2018, which led to an exponential increase in the number on ARTs. In addition to the large number of those on ARTs, South Africa has experienced a steady increase in the number of patients with noncommunicable diseases (NCDs) who required chronic therapy.^1,4,5,6^

The resultant high volume of patients accessing healthcare facilities has adversely affected service delivery, with healthcare workers (HCWs) at highly congested facilities experiencing high levels of stress and fatigue, resulting in a high staff turnover and absenteeism rates, staff shortages and substandard patient care. These factors often result in reduced attention by HCWs to issues of patient compliance and hence treatment failure. For patients, the negative consequences of congested facility include dissatisfaction; long waiting times; increased loss to follow-up; repeat visits to get attended to, which results in unexpected transport costs; and loss of income on the days that they (re)attend the clinic.^6,7^ The Department of Health has to contend with ineffective disease control and increased burden on the healthcare facility’s infrastructure, as evidenced by overcrowded waiting area space for patients and insufficient storage space for the resultant large quantities of chronic medication and ARTs required.^1,6,8,9^

To provide quality care to a large number of patients without increased resources, HCWs needed to adopt a chronic disease model that entailed task shifting and finding innovative measures to distribute and dispense ART and other chronic medication. These initiative measures included, (1) issuing of pink card as part of ‘routine’ care and (2) implementing the Central Chronic Medicines Dispensing and Distribution (CCMDD) programme. During routine care, after assessment, a stable patient who is meeting treatment targets and has no complications that need active management is issued with a repeat prescription for 6 months (a ‘pink card’), which enables them to attend the healthcare facility monthly to only collect medication. Unfortunately, the ‘pink card’ only allows for 1 month dispensary of medication and patients must present to the healthcare facility for a twice-yearly review. On a daily basis, as much as 70% of a facility’s prescription load is devoted to renewing repeat prescriptions.^6,10,11^

The CCMDD programme began in February 2014 and was developed as a strategy to decongest healthcare facilities and improve service delivery by promoting patient’s access to their chronic medication by allowing them to collect their medication at the nearest convenient pick-up point (PUP) such as the local post office or other convenient places, for example, at Clicks, rather than at the clinic. The programme is active in more than 46 districts (88%) within the public sector, and by 2020, over 73% of patients had been enrolled.^6,12^ It was anticipated that the programme would (1) improve patient experiences by reducing (or eliminating) waiting times, (2) make medication collecting easier because of the extended operating hours at some PUPs, and (3) reduce lost to follow-up because of the greater convenience of collection and reduced HCW discrimination.

Patients on the CCMDD programme have all their relevant information captured on tier.net system, a national database that includes patient’s demographics as well as blood results since they have started antiretroviral treatment. HIV-positive patients are eligible for the CCMDD programme if they had been clinically stable for 6 months, compliant to their medication, and have an undetectable viral load (VL). In addition, all patients are assumed to be compliant to their treatment for 6 months until their next review, with SMS notifications informing them when and where to collect their medication.

At the time of the study (01 January 2018 – 31 December 2018), Medipost, the pharmaceutical service provider, was responsible for providing medication to patients registered onto the CCMDD programme. Medipost was also responsible for ensuring that the pre-packed medication parcels were delivered to the various PUP collection points around the city 48 h before the patient’s due date. In addition, Medipost sent a text message to the patient reminding them to collect their medication from the chosen PUP once it had been delivered. Should the medication not have been collected within 2 days, then Medipost sent the patient an additional text message as a reminder to collect it. If the medication was not collected within 14 days, Medipost sent the community health centre (CHC) a list of patients who did not collect their medicines and a defaulter list was submitted for capture into the tier.net system to update the patients’ compliance status.^6,13,14^ If the medication was not collected after a further 2-week period, it was sent back to the depot and the CHC concerned was informed of the missed appointment, which then activated staff at the CHC to track and trace the patient to prevent their defaulting.^6,13,15,16^

For the CCMDD programme to work efficiently, healthcare facilities, including the East Boom CHC (EBCHC), gave patients dates for monthly medication collection and for relevant blood sample collection at month 5 (fast queue). The patients were given the date to visit the CHC for a 6-month follow-up, review of their results and a repeat script. In addition, the EBCHC housed all patient files and ensured that an active tracing system was in place for those who defaulted. Patients were reviewed every 6 months and diverted back to the PUPs for medication collection if they remained clinically and virologically stable.^6,17,18,19^

Those patients who had an increased VL (> 150) result when their bloods were reviewed or other factors that required them to be more regularly monitored for routine care, attended the facility on a monthly basis and collected medication from the pharmacy.

There has been concern that the limited amount of contact time between patients on the CCMDD programme and HCWs could have a negative impact on compliance, and in the long term, control of the HIV pandemic. This study aimed to compare patient satisfaction and virological suppression among those who collected medication through the CCMDD programme and routine care.

## Research methods and design

### Study design

An observational, retrospective analytical study was conducted at the EBCHC, which compared patients who collect their ARTs monthly from the CHC with those who did so at an external PUP via the CCMDD programme. East Boom CHC was purposively selected because of the large number of patients attending this facility for the ART programme – approximately 12 000 patients and the largest patient population within the UMgungundlovu District.

### Study setting

East Boom CHC is centrally situated within the central business district of Pietermaritzburg. There is no defined catchment area for this healthcare facility, as the facility is easily accessible via taxi and bus routes. Patients attending the facility are mainly from urban areas; however, the facility is flanked by two large informal settlements that house patients who are unemployed.

### Data collection

The study was conducted over 12 months, from 01 January 2018 to 31 December 2018, during which time the CCMDD programme became a mandatory monitoring indicator for managers as a tool to monitor the number of patients who were eligible for the CCMDD programme versus those were actively registered on the programme. Patients in the CCMDD group attended the healthcare facility twice within a 12-month period, while those attending the routine care group attended monthly to collect their medication.

### Population, sampling technique and sample size

The study population were patients who were 18 years and older, clinically and virologically stable (VL < 40 copies [cps]/mL or lower than detectable), on Regimen 1 (Fixed Drug Combination at the time of study: tenofovir disoproxil fumarate (TDF)/emtricitabine (FTC)/efavirenz (EFV) at the time of study) and who collected their treatment from the EBCHC or via the CCMDD programme.

A sample size of 117 patients from each group were chosen (234 patients) – this is equivalent to detecting a medium effect size (difference) between the two groups using a repeated measure analyses of variance (ANOVA). Group sample sizes of 117 each achieve 80% power to detect a difference of 0.300 in a design with 3 repeated measurements having a compound symmetry covariance structure when the standard deviation is 1.000, the correlation between observations on the same subject is 0.500, and the alpha level is 0.050.^13^

The first 20 patients from each group who presented each day at the EBCHC and who met the inclusion criteria were invited to participate in the study until the study sample size was reached (117 collecting medication from the CHC and 117 on the CCMDD programme). All eligible patients were provided with information on the study in IsiZulu (the home language of most patients) or English. Informed consent was obtained from those willing to participate and a questionnaire was completed at the time of selection (T0), 6- and 12-month reviews. Unique patient identifiers were used to ensure anonymity and to link their questionnaire responses over time and with their profiles on tier.net. Patients self-completed the questionnaire and were helped by staff members if they were illiterate. The questionnaire included demographic data (age, gender, marital status, education level) and occupation (student, self-employed, pensioner, formally employed, unemployed), including subjective indicators, such as *patient satisfaction with their respective services*, consumption of traditional medication for health purposes, excessive alcohol consumption and if they used condoms consistently over the last 6 months since the previous review. These terms were not defined and patients were asked to choose between always, sometimes or never in response to these questions. Data on their duration of treatment and VLs were accessed from the national medicines database, tier.net, as well as whether or not they collected their medication on time during the study period.

### Data analysis

Data were captured using EpiData and summarised using descriptive methods, with a comparative analysis of the two groups.

### Ethical considerations

Ethical clearance to conduct this study was obtained from the University of KwaZulu-Natal, Biomedical Research Ethics Committee (No. BE315/16) as well as from the administration of East Boom CHC. The survey was anonymous and the participants were assured of confidentiality.

## Results

All patients agreed to participate in the study, and 117 patients from the routine group and 117 patients from the CCMDD programme completed the questionnaire at T0, 6 months and 12 months. Patients’ demographics are presented in Table 1, which shows that more females (56%) participated than males (44%). Participants’ ages ranged from 22 years to 57 years, with a mean age of 47 years. Those in routine care were younger than those in the CCMDD programme, and the mean duration of years on treatment was 3 years (range: 1–6 years).

**TABLE 1:** Patients’ demographics at baseline.

Study groups	Total		Males		Females		Mean age (years)		Mode (years)	Mean duration on ART (years)	
	*n*	%	*n*	%	*n*	%	Mean	Range		Mean	Range
Routine care	117	50	49	42	68	58	38	22–57	38	3	1–6
CCMDD	117	50	53	45	64	55	42	22–57	39	3	1–6
**Total**	**234**	**100**	**102**	**44**	**132**	**56**	**47**	**22–57**	**46**	**3**	**1–6**

CCMDD, Central Chronic Medicines Dispensing and Distribution; ART, antiretroviral therapy.

Table 2 shows that the routine care group began with 117 patients at baseline and only 29% remained in the group at the 6 months review, with 100% having transitioned into the CCMDD group at 12 months. All patients had a VL < 40 cps/mL at the beginning of the study, this being an inclusion criterion, which was maintained throughout the year.

**TABLE 2:** Retrospective analysis of patients in the routine care group.

Total number of patients	Frequency (*n*)	Mean viral load (copies/mL)
Baseline	117	< 40
6 months review	34	< 40
Number transitioned to CCMDD group	83	< 40
12 months review	0	-
Number transitioned to CCMDD group	117	< 40

CCMDD, Central Chronic Medicines Dispensing and Distribution.

Table 3 shows that all patients in the CCMDD group had a VL < 40 cps/mL (inclusion criterion) at baseline. All patients from the routine group transitioned to CCMDD during the course of the study (*n* = 83 [71%] by 6 months and all 117 [100%] by 12 months). All of the patients with VL > 40 cps/mL came from the routine care group.

**TABLE 3:** Retrospective analysis of patients in the Central Chronic Medicines Dispensing and Distribution programme group.

Total number of patients	Frequency (*n*)	Mean viral load (copies/mL)
Baseline	117	< 40
6 months review	200	< 40
Number transitioned to CCMDD group	0	-
Number transitioned to routine group	83	< 40
12 months review	234	†
Number transitioned to CCMDD group	0	-
Number transitioned to routine group	34	< 40

CCMDD, Central Chronic Medicines Dispensing and Distribution programme.

† , 227 had VL < 40 copies/mL; 7 had VL > 40 copies/mL but < 1000 copies/mL.

Table 4 shows data received on patients who had elevated VL levels.

**TABLE 4:** Patients with elevated viral loads.

Variable	< 40 copies/mL	50–100 copies/mL	100–500 copies/mL	500–1000 copies/mL	> 1000 copies/mL
Patient details	100 male patients	1 female (65 cps/mL) from routine care	1 male (223 cps/mL) from routine care	1 female (584 cps/mL) from routine care	Nil
-	127 female patients	1 female (86 cps/mL) from routine care	1 male (104 cps/mL) from routine care	-	-
-	-	-	1 female (315 cps/mL) from routine care	-	-
-	-	-	1 female (120 cps/mL) from routine care	-	-
**Total**	**227**	**2**	**4**	**1**	**234**

cps, copies.

**FIGURE 1:**
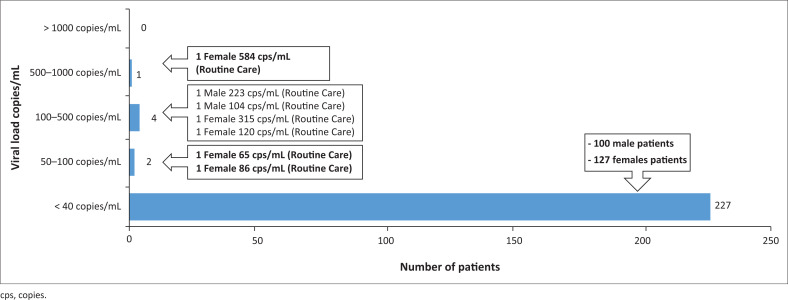
Analysis of patients with elevated viral loads.

Figure 1 shows that 7 (3%) patients had increased VLs, while 227 (97%) maintained their levels of < 40 cps/mL, the highest being 584 cps/mL for a female. Six females had raised VLs versus one male, all those with raised levels being original patients of the routine care group.

Table 5 highlights the concerns of those patients in the routine care group who had to wait in long queues to see the healthcare professionals, while the CCMDD participants did not indicate the problems with accessing their medication.

**TABLE 5:** Analysis of viral load of all patients at the end of 12 months.

Viral load	Males	Females
< 40 cps/mL (*n* = 227)	100	127
50–100 cps/mL (*n* = 2)	-	1 (65 cps/mL): routine care 1 (86 cps/mL): routine care
100–500 cps/mL (*n* = 4)	1 (223 cps/mL): routine care 1 (104 cps/mL): routine care	1 (120 cps/mL): routine care 1 (315 cps/mL): routine care
500–1000 cps/mL (*n* = 1)	-	1 (584 cps/mL): routine care
> 1000 cps/mL (*n* = 0)	-	-
**Total (*n* = 234)**	**102**	**132**

cps, copies.

Table 6 identifies the major complaint that emanated from routine care patients, which was long waiting times. Similarly, Table 6 also displays the movement of patients from the routine care group to the CCMDD group.

**TABLE 6:** Patient satisfaction survey.

Patients	Number	Complaints
Routine care at 6 months review	34	Long waiting times
Routine care at 12 months review	0	0 patients
CCMDD at 6 months review	200	0
CCMDD at 12 months review	234	0

CCMDD, Central Chronic Medicines Dispensing and Distribution.

Table 7 reports on other important aspects of care with all patients reporting using of condoms, no use of traditional medication or excess alcohol and compliance with their treatment.

**TABLE 7:** Subjective data obtained from the retrospective study.

Factors	Routine care at baseline	Routine care at 6 months	Routine care at 12 months	CCMDD at baseline	CCMDD at 6 months	CCMDD at 12 months
Used condoms	117	34	0	117	200	234
Used traditional medication	0	0	0	0	0	0
Used alcohol	0	0	0	0	0	0
Treatment compliant	117	34	0	117	200	234

CCMDD, Central Chronic Medicines Dispensing and Distribution.

**FIGURE 2:**
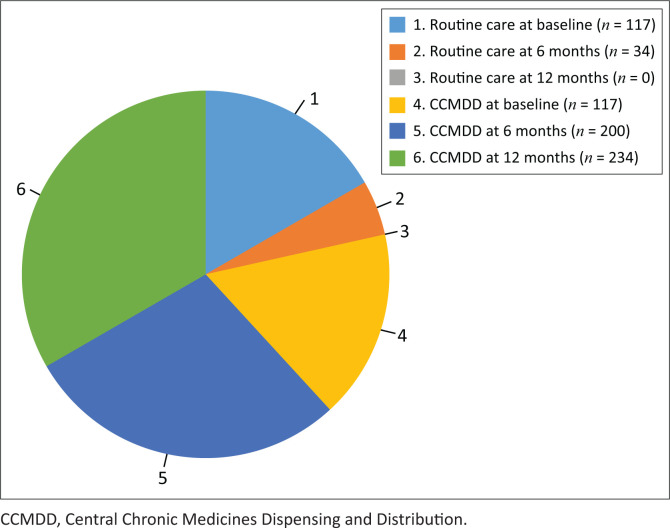
Comparison of factors across different stages of care and treatment compliance.

Figure 2 is an illustration of the data obtained from Table 7, which shows the movement of patients from the routine care group into the CCMDD group. Patients throughout the study alleged compliance to medication, compliance to the use of condoms, and no ingestion of alcohol and traditional medications.

## Discussion

The study focused on monitoring and comparing the patients from two ART groups: routine care (monthly visits to the health facility to collect medication) and CCMDD (medication collection at a PUP) over a 12-month period in an urban setting. The participants were mainly young and economically active, this being consistent with another study that reviewed medication uptake in the CCMDD programme elsewhere in South Africa.^20^ Despite comparable infection rates, there were more women than men in this study. Numerous studies indicate the gender distribution that shows more women than men receive ARTs.^20^ This is likely because of a number of factors, including health-seeking behaviour, and emphasises the need to find ways to ensure that men who test HIV-positive are able to access services earlier.

Regarding the patients in the routine care group (Table 2), the results showed that all patients moved out of the routine care group and into the CCMDD programme, which was only possible when their VLs were < 40 cps/mL. Possible reasons for this include frustration with the long waiting times at the clinic as highlighted in Table 6, the convenience offered by the CCMDD programme, and the encouragement of HCWs who wanted to reduce the patient numbers of those who only needed to collect ART and chronic medication. A study by Bassett et al.^20^ showed that factors that improved the CCMDD uptake were the opening hours of some PUPs (e.g., weekends), their convenient locations and not waiting in long queues for those who were employed. These findings were similar to those of Bogart et al.^17^ and Liu et al.^13^ and highlight the importance of providing a service that is accessible and convenient for patients who are working.

A total of 227 patients remained virologically suppressed (97%) while 7 had VLs higher than 40 cps/mL, all being from the routine care group, and not being an expected outcome. Routine care was thought to be more advantageous than the CCMDD programme because of the patients receiving health education at each visit while waiting to collect their medication. The seven patients were deregistered from the routine care programme and received additional compliance counselling with their scheduled monthly follow-up visits. Their VLs were repeated after 3 months, and once they were virologically suppressed, they were transferred onto the CCMDD programme.

This shows the ability of the system to detect patients whose VLs are not adequately suppressed. As per the National ART Guidelines,^1^ additional compliance counselling was provided and patients were able to continue on their current regimen. As compliance was considered the main reason for the slightly high VL, staff considered transfer to the CCMDD programme as appropriate only when their VL was less than 40 cps/mL. Other studies have found that when patients are transferred onto the CCMDD programme, patient’s compliance improves and their VL goes down.^20^

Regarding the participant’s VLs (Table 3), all those in the CCMDD programme maintained a VL < 40 cps/mL. Despite the concern that without constant reinforcement from HCWs, patient compliance to ARTs would decline,^1^ the CCMDD programme demonstrated that they can maintain their virological suppressed status while collecting medication from external PUPs and only having clinical contact every 6 months. It is important to understand what is communicated at the six-monthly visits to ensure good compliance to ART. Along with other studies, this study showed that the CCMDD programme can be used as an effective means to decongest healthcare facilities for eligible patients, as indicated in the studies by Bogart et al.^17^ and Liu et al.^13^ All patients in both groups reported excellent compliance to medication (Table 7), a claim supported by the low VLs recorded in the vast majority. Although there is some uncertainty about the accuracy of the data from the questionnaire, the fact that all patients in the CCMDD programme had VL < 40 cps suggests that they were in fact compliant with their medication. In addition, all patients reported consistent condom usage, no alcohol consumption and no use of traditional medication. The always and/or sometimes and/or never format and having to remember what happened over the last 6 months was a limitation of the questionnaire and made it difficult to interpret the data, as it is unlikely that 100% of participants never abused alcohol, never took traditional medication and used condoms every time they had sex. Patients are often subject to abuse from staff at the clinic if they admit to alcohol use, concomitant use of traditional medication and not condomising. It does seem unlikely that these responses are accurate and may be a reflection of their unwillingness to give correct answers for fear of being reprimanded by the staff, despite being informed that their responses were confidential. In addition, these are standard questions that patients are routinely asked when they come for review, and it is unusual for a patient to ever admit that he or she is not compliant with these instructions given by the staff. This needs further investigation to determine the correct use of condoms and traditional medication so that strategies can be developed to address these issues rather than accepting these results and congratulating ourselves on a job well done.

This report of excellent compliance to medication and the use of condoms as well as abstaining from alcohol and traditional medication is encouraging as healthcare workers assumed that with infrequent health eduction, patients may revert to poor treatment compliance modalities. Other studies have also shown continued compliance with these lifestyle messages when patients are on the CCMDD programme.^13,17^ It would appear that good initiation programmes covering issues such as the importance of compliance, condom usage, no traditional medication and excess alcohol consumption are more important than regular (and often inconvenient) clinic visits.^17^ However, a small number of patients had raised VLs, with poor compliance being the most common reason, the likelihood being that not all patients were 100% compliant, despite what they reported in the questionnaire. This may be because of fear of reporting poor compliance in case they were referred back to monthly clinic care.

The results of this study show that those stable patients who collected their medication from external PUPs remained virologically suppressed. The fact that 100% of patients chose to move from routine care into the CCMDD programme highlights that they prefer the convenience of collecting their treatment from PUPs and do not need to attend monthly clinic visits to remain compliant to their ARTs and virologically suppressed.

A number of limitations may have affected the findings, these being that the data were only collected at one healthcare facility in Pietermaritzburg, KwaZulu-Natal – an urban area, and may not therefore be applicable to rural chronic patients, where access to PUPs may be limited. Memory recall bias, or the desire to give the presumed correct responses, may have influenced the questionnaire results.

### Recommendations

There should be more studies on the effectiveness of the CCMDD programme to determine if the review time could be extended to 12 months and to evaluate and implement modalities to improve health-seeking behaviour of patients, especially men.

## Conclusion

The study shows patient satisfaction with the CCMDD programme, which is the preferred option, and most patients maintain VL stability. This suggests that the CCMDD programme is a viable solution for decongesting healthcare facilities by allowing stable patients to collect medication from PUPs. The CCMDD programme has the potential to be scaled up to enable patients to be reviewed annually instead of every 6 months.
